# The feasibility and safety of adopting the left lumbar vein to localize the renal artery location during left transperitoneal laparoscopic partial nephrectomy

**DOI:** 10.3389/fsurg.2022.858798

**Published:** 2022-09-05

**Authors:** Zhongshun Yao, Jiming Zhao, Bin Zheng, Zixiang Cong, Yiming Zhang, Jiaju Lv, Zhihong Niu, Fajuan Cheng, Wei He

**Affiliations:** ^1^ Department of Urology, Shandong Provincial Hospital Affiliated to Shandong First Medical University, Jinan, China; ^2^ Department of Urology, Shandong Provincial Hospital Affiliated to Shandong University, Jinan, China; ^3^ Department of Nephrology, Shandong Provincial Hospital Affiliated to Shandong First Medical University, Jinan, China; ^4^ Department of Nephrology, Shandong Provincial Hospital Affiliated to Shandong University, Jinan, China

**Keywords:** laparoscopy, lumbar vein, renal cell carcinoma, transperitoneal approach, surgery

## Abstract

**Background:**

Laparoscopic partial nephrectomy (LPN) is the standard of care for localized small renal cancer. The most critical step in this form of surgery is to localize the renal artery. In the present study, we describe a novel technique that uses the left lumbar vein (LV) to access the left renal artery during LPN.

**Materials and methods:**

This was a retrospective review of 130 cases of transperitoneal laparoscopic partial nephrectomies (TLPNs) performed on patients with renal cancer in our center between January 2018 and December 2021. Either the LV or non-lumbar vein (N-LV) technique was used to locate and manage the left renal artery. We recorded relevant clinical data from all patients, including patient characteristics, tumor data, and perioperative outcomes (artery mobilization time, operative time, estimated blood loss, and complications). Comparative analysis was then carried out between the cases using LV or N-LV vein techniques.

**Results:**

All TLPNs were successfully accomplished without conversion to open approaches. There were no complications involving the renal vessels during the entire study. The LV technique resulted in a significantly shorter time to mobilize the renal and significantly less estimated blood loss (*p* < 0.05). There was no significant difference between the two techniques with regard to perioperative complications.

**Conclusion:**

The left LV represents an anatomical landmark for locating the left renal artery in TLPN. This approach has numerous advantages over the transperitoneal approach including facilitating access to the left renal artery and reducing the duration of surgery.

## Introduction

The contemporary surgical management of renal cancer has undergone a dramatic change over the last few decades. Clayman et al. were the first to report transperitoneal laparoscopic nephrectomy at Washington University in 1990 (1). Since then, long-term clinical data have confirmed that laparoscopic surgery performed in well-selected patients yields oncological results that are similar to that of traditional open surgery (2). In addition, growing experience and advances in both instruments and devices have led to the increasing adoption of laparoscopy worldwide. Laparoscopic partial nephrectomy (LPN) is the standard of care for small renal cell carcinoma (3). While renal laparoscopy can be performed with a transperitoneal or retroperitoneal approach, transperitoneal access has been preferred due to the large working space and the presence of familiar and identifiable anatomical landmarks (4). Although the overall technique for renal transperitoneal laparoscopy is well established, it remains a challenge for a newcomer. The most vital part of this surgery is the localization of the renal artery. Herein, we describe a novel technique for locating the left renal artery during transperitoneal laparoscopic partial nephrectomy (TLPN). The main intention to describe this new technique is to help novice urologist to secure the position of the left renal artery when performing TLPN on the left kidney.

## Materials and methods

**Figure 1:**
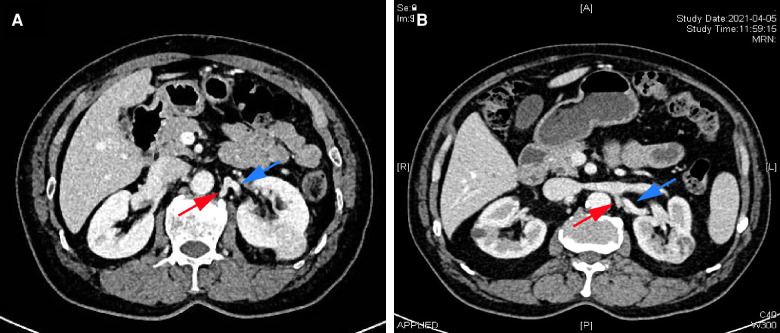
Perioperative computed tomography (CT) scans from two patients with renal cancer patients. (**A,B**) Renal contrast-enhanced CT demonstrating the lumbar vein (red arrow) and the left renal artery (blue arrow).

This study was conducted on renal cancer patients who underwent TLPN performed by one urologist (Dr. Niu) between January 2018 and December 2021. Dr. Niu is an expert laparoscopic surgeon who performs at least 200 laparoscopies annually (including adrenalectomy, partial nephrectomy, radical nephrectomy, radical cystectomy, and radical prostatectomy). After approval by the Institution Review Board (SWYX: NO.2022-012), we retrospectively re-evaluated preoperative computed tomography (CT) images and videos recorded during surgery. All patients met the following criteria: (1) the initial diagnosis of primary resectable left renal cell carcinoma, (2) the left lumbar veins (LVs) were identified from the perioperative CT scan (Figures 1A,B), (3) the presence of a single clinical localized renal tumor (cT1a) eligible for partial nephrectomy, and (4) the patient underwent TLPN. The exclusion criteria were: (1) T1b, T2, or left local advanced renal cell carcinoma with tumor thrombus or hilar structure invasion, (2) preoperative CT demonstrated the absence of the left LV converging with the left renal vein, (3) a history of abdominal surgery, and (4) the lack of videos recorded in surgery. A total of 130 qualified patients were analyzed in this study. Based on whether the left LV was used as an anatomical marker to locate and dissect the left renal artery, the patients were separated into two groups: the LV group and the non-lumbar vein (N-LV) group.

The patients were positioned in a right 70° lateral decubitus position with a kidney bridge elevated. Peritoneal access is obtained by either the Veress-needle technique or the Hasson cannula technique, as described previously (5). Typically, a three-trocar technique was usually employed. A 10-mm port was inserted at the lateral border of the rectus muscle 2 cm cephalad to the umbilicus (camera port); a 5-mm port was placed at the mid-clavicular line near the costal margin. Another 12-mm port was inserted at the mid-clavicular line 2 cm above the anterior superior iliac spine.

**Figure 2:**
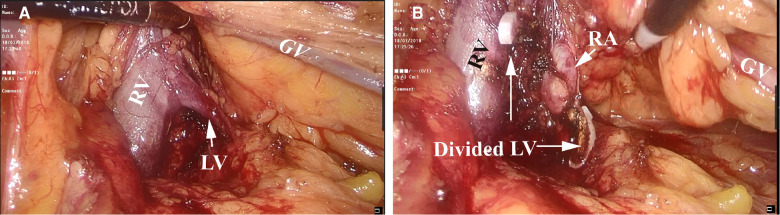
Mobilization and identification of the left renal artery. (**A**) Tracing of the left gonadal vein (GV) leading to the left renal vein (RV) and the left lumbar vein (LV). (**B**) After securing and dividing the left LV, the left renal artery (RA) was brought into clear view.

The line of Toldt was incised from the level of the iliac vessels to the superior pole. The incision was further performed cephalad to the splenorenal ligament and the splenodiaphragmatic attachments, freeing both the spleen and the colon. Then, the descending colon and its mesentery were dissected from the Gerota's fascia. The splenocolic ligament was incised, thus allowing the spleen and colon to reflect medially. After colonic reflection, the Gerota's fascia entered parallel to the aorta. Blunt dissection between the retroperitoneal fat and psoas muscle was then used to identify the gonadal vein and ureter. The gonadal vein was then traced cephalad to its insertion into the left renal vein. The perirenal fat surrounding the lower pole of the kidney was gently mobilized, retracted, and twisted anterolaterally. In the LV group, careful dissection along the left renal vein allowed the identification of the LV. After the LV had been secured and divided, the left renal vein was retracted medially to bring the renal artery into clear view (Figures 2A,B). The renal artery was then circumferentially mobilized. In the N-LV group, we first dissected the lower pole of the kidney and then identified the hilum. Then, the left renal artery was completely mobilized behind the renal vein. The main renal artery clamping strategy was used to block kidney perfusion in the present study. Next, partial nephrectomy was performed, as described previously (6, 7).

We used the interval between entering the Gerota's fascia and the satisfying exposure of the left renal artery as an objective index. Patient demographics, clinical features, blood loss, and other perioperative parameters, were recorded and analyzed. Continuous variables between the two groups were compared with the Mann–Whitney test. Categorical variables were analyzed using the chi-squared test to determine significant differences. A two-tailed *p-*value <0.05 was considered as statistically significant. Statistical analyses were performed with SPSS 22.0.

## Results

Patient characteristics are summarized in Table 1. Overall, TLPN was performed on 130 patients. Most of the patients were male (72.3%, 94/130). The mean age of the patients was 57.4 years, and the mean body mass index (BMI) was 25.8 kg/m^2^. The renal tumors were located on the ventral surface of the middle part of the left kidney (*n* = 57, 43.8%) or the lower pole (*n* = 73, 56.2%). Seventy-three patients were treated with the LV technique (LV group, 73/130, 56.2%). Fifty-seven patients were treated without the LV technique (N-LV group, 57/130, 43.8%). The diameters of the tumors in the LV group were slightly smaller than those in the N-LV group (median, 3.2 cm vs. 3.5 cm) and were not statistically significant (*p *= 0.158). No statistical significance was observed concerning the RENAL score (*p *= 0.44). The New York Heart Association (NYHA) and American Society of Anesthesiology (ASA) score systems are summarized in Table 1 without significance (*p *= 0.297 and *p *= 0.560).

**Table 1:** Patient characteristics.

Characteristic	LV (*n* = 73)	N-LV (*n* = 57)	*p*-Value
Age, years	58.5 ± 10.2	56.0 ± 13.0	0.209
Sex (*n*)			
Male	55 (75.3%)	39 (68.4%)	0.382
Female	18 (24.7%)	18 (31.6%)	
BMI, (kg/m^2^)	26.0 ± 2.8	25.4 ± 2.9	0.275
Tumor size (cm)			
Median, range	3.2 (2.5–3.7)	3.5 (2.7–3.8)	0.158
Tumor location [*n* (%)]			
Ventral surface of the middle part	37 (50.7%)	20 (35.0%)	0.823
Below the lower pole line	46 (49.3%)	37 (65.0%)	
RENAL risk group			
Low complexity (score 4–6)	58 (79.5%)	42 (73.7%)	0.44
Moderate complexity (score 7–9)	15 (20.5%)	9 (26.3%)	
NYHA classification			
I	51 (70.0%)	35 (61.4%)	0.297
II	21 (28.6%)	22 (38.6%)	
III	1 (1.4%)	0	
ASA score			
I	1 (1.4%)	0	0.560
II	58 (79.6%)	46 (86.8%)	
III	14 (19.0%)	11 (13.2%)	

ASA, American Society of Anesthesiology; BMI, body mass index; LV, lumbar vein; N-LV, non-lumbar vein; NYHA, New York Heart Association.

The operative outcomes are outlined in Table 2. In all patients, we were able to identify the left LVs from the perioperative CT scan. The majority of patients had one LV. The mean operative time for the patients who received the LV technique was 115 min; this compared to 125 min for patients receiving the N-LV technique, there was no significant difference between the two groups with this respect (*p* = 0.035). The time taken to mobilize the artery in the LV group was significantly shorter than that in the N-LV group (15  vs. 19 min, *p* = 0.001). Furthermore, there was a significant difference between the two groups with regard to estimated blood loss (*p *= 0.037). No statistically significant differences were identified between the two groups regarding surgical drainage and postoperative hospitalization. The postoperative complications are outlined in Table 2 without significant differences between the two groups. Four cases were diagnosed with lower limb deep vein thrombosis. Three patients had an acute cardiovascular or cerebrovascular accident. Five patients suffered a wound infection. Urinary leak was observed in two patients. All of them recovered after conservative treatment. No perioperative deaths occurred.

**Table 2:** Perioperative data.

Characteristic	LV (*n* = 73)	N-LV (*n* = 57)	*p*-Value
Number of lumbar veins			
One	70 (95.9%)	53 (92.9%)	0.736
Two	3 (4.1%)	4 (7.1%)	
Number of renal arteries			
One	58 (79.4%)	47 (82.5%)	0.706
Two	15 (20.6)	9 (15.8%)	
Three	0	1 (1.7%)	
Operative time			
Median, range	115 (105–140)	125 (115–150)	0.035*
Artery mobilization time			
Median, range	15 (14–17)	19 (17–23)	<0.001*
EBL (ml)			
Median, range	50 (40–60)	50 (40–80)	0.037*
Day to surgical drain removed			
Median, range	3 (3–3)	3 (3–4)	0.083
Postoperative hospital stay (days)			
Median, range	4 (4–5)	4 (4–5)	0.531
Pathologic type [*n* (%)]			
ccRCC	64 (87.7%)	51 (89.5%)	0.873
Others	9 (12.3%)	6 (10.5%)	

Artery mobilization time, the interval between entering the Gerota's fascia and success exposure of left renal artery; EBL, estimated blood loss; ccRCC, clear cell renal cell carcinoma.

*p < 0.05.

Pathological analysis of the tumors is summarized in Table 2. There was a predominance of clear cell renal cell carcinoma (RCC) in each group.

## Discussion

Over the last 20 years, laparoscopic surgery has become the standard treatment for renal cancer. Transperitoneal and retroperitoneal approaches are widely used to perform minimally invasive surgery. Transperitoneal surgery has the advantages of large surgical spaces and identifiable anatomical landmarks. However, the identification and mobilization of the renal artery are crucial for the transperitoneal laparoscopic approach. Typically, the ureter or gonadal vein is traced cephalad from the iliac vessels towards the kidney. The ureter ends in the renal pelvis just beneath the renal vein. In a manner that is different from the right side, the left gonadal vein converges with the left renal vein, thus making it easier to locate the left renal vein. Once the renal vein has been identified, the renal artery can be isolated behind it (8). Compared to the retroperitoneal approach, the renal artery is often found behind the renal vein; this makes it difficult to identify transperitoneally. Only a few studies have focused on the transperitoneal technique for positioning the renal artery.

Porpiglia et al. reported their experience of direct access to the renal artery at the level of the Treitz ligament for left side nephrectomy (9). These authors made an incision in the Treitz ligament and posterior peritoneum along with the inferior mesenteric vein. After the anterior-lateral surface of the aorta was identified, the dissection continued cephalad. Once the renal vein which crossed over the aorta, was retracted, the renal artery was identified and carefully dissected up to its origin. In another, Tunc et al. (10) reported a modified technique with rapid access and early ligation of the renal pedicle. These authors detached the upper pole of the kidney from the surrounding structures. Renal hilar structures, including the renal artery and vein, were then identified directly. The renal pedicle was ligated and cut *en bloc*. Thereafter, the kidney was dissected, and the ureter was identified and cut. The authors suggested that this modified technique facilitated laparoscopic nephrectomy in terms of operation time. Zhang et al. (11) described their experience with direct lateral access to the renal artery for transperitoneal partial nephrectomy. The perirenal fat was dissected at the lower pole of the kidney. The ureter and gonadal veins were then identified and retracted anterolaterally. After the pulsation of the renal artery was observed, the renal artery was then exposed to an electric hook or harmonic.

The LVs are segmentally arranged retroperitoneal vessels that receive blood from the posterior abdominal wall and the paravertebral muscles. Several studies have investigated the exact pattern of this lumbar vasculature (12–14). Knowledge of the LV is important for urologists when operating in the transperitoneum. Comparably, the renal venous pattern on the left side has little resemblance to that on the right. The longer left renal vein regularly receives several tributaries: suprarenal, gonadal (testicular or ovarian) from below, and the LV posteriorly (15). Given the complexity of the left renal vein and its tributaries, it is more challenging to identify the left renal artery transperitoneally. If the LVs are divided without reliable ligation, they may retract into the paravertebral tissue with the risk of serious venous hemorrhage. As the “tricky” LVs communicate with the left renal vein across the left renal artery, they are crucial not only for preventing bleeding by accidental tearing but also for orienting to the left renal artery. In the present study, we used the left LV as a landmark to locate the left renal artery during transperitoneal surgery.

Following mobilization of the descending colon and its mesentery away from Gerota's fascia, the perirenal fascia was incised parallel to the aorta. The space between the lower pole of the kidney and the psoas muscle was expanded to the renal pedicle. The left renal vein was dissected carefully. Then, the LVs draining into the left renal vein were identified and divided with Hem-o-lok™. The left renal vein can be retracted more anteriorly, thus providing a better angle and enhancing visualization of the posterior wall of the left renal vein; this allowed clear identification of the renal artery. In the present study, artery mobilization time was defined as the interval between entering the Gerota's fascia and the successful exposure of the left renal artery. There was no difference between the two groups with regard to tumor complexity based on the RENAL nephrometry score. Although we did not find any significant difference between our two groups of patients in terms of surgical drain removal and hospital stay Furthermore, the utility of the left LV as an anatomical landmark facilitated the TLPN by reducing the mobilization time of the left artery and intraoperative blood loss when compared to the control group.

The advantages of this technique can be summarized as follows. First, mobilizing and dividing the left LV in advance can prevent unexpected bleeding. Second, this technique is time-saving as it does not involve the mobilization and rotation of the laterodorsal part of the kidney to locate the left renal artery. Third, after the communicating left LV is divided and ligated, the left renal vein can be retracted much more anteriorly to remove the “crimping” wrapping effect of the left renal vein, thus facilitating exposure to the origin of the left artery from the aorta. This is vital for total control of the left renal artery, avoiding branches omitting. Clinically, we adopt different clamping strategies during part nephrectomy, including off-clamp, selective clamp, and main renal artery clamp. Off-clamp is usually used for small exophytic renal mass avoiding renal artery location. Selective renal artery clamp is often performed for hilar renal tumors. To investigate the feasibility of left LV to access the left renal main artery, we have used the main renal artery clamp in both groups. Finally, the technique reduces the risk of cancer cell spread as manipulation of the renal tumor is reduced when it locates laterodorsally.

To the best of our knowledge, this is the first study to focus on the left LV as an anatomical landmark for left kidney transperitoneoscopic surgery. In our experience, this technique appears to facilitate this type of surgery without increasing the incidence of perioperative complications.

The present study had some limitations that should be noted. First, this study was conducted in a retrospective manner; thus, we cannot avoid selective bias. Second, our study did not consider whether the LV is descending, ascending, or a variation of the venous ring around the aorta. The high variability of these vessels makes it very difficult to provide precise information. However, the technique can help the urologist focus on the drainage point of the lumbar vessels into the left vein and association with other tributaries when performing transperitoneal laparoscopic approaches to the left renal artery. Finally, although statistical differences were observed in terms of artery mobilization time and blood loss, the sample size was small. Further studies, with larger sample sizes, are now warranted to validate these preliminary findings.

## Conclusion

The findings of the present study suggest that the left LV can be used as an anatomical landmark to locate the left renal artery during TLPN. This technique offers the advantages of the transperitoneal approach, facilitates access to the left renal artery, and reduces operation time and blood loss.

## Data Availability

The original contributions presented in the study are included in the article/Supplementary Material, further inquiries can be directed to the corresponding authors.
